# Rebalancing Life: A “MOBILE” Model for Addressing Mood Instability and Occupational Challenges in MDD

**DOI:** 10.1192/j.eurpsy.2025.860

**Published:** 2025-08-26

**Authors:** S. Volovic Shushan, A. Stern, N. Josman

**Affiliations:** 1 The Faculty of Social Welfare and Health Sciences, Department of Occupational Therapy, University of Haifa, Haifa; 2 “Clalit” health services, “Shalvata” Mental Health Hospital, Hod-Ha’Sharon; 3 Faculty of Health Sciences, Occupational Therapy Department, Ben-Gurion University of the Negev, Beer Sheva, Israel

## Abstract

**Introduction:**

Major Depressive Disorder (MDD) is one of the most well-known causes of disability worldwide. It is characterized by a persistent pattern of low mood, low self-esteem, and loss of interest and pleasure, leading to extensive functional impairment. Another characteristic is emotional dysregulation, leading to mood instability (MI), a frequent and intense fluctuations in emotions over time. MI affects the daily function of people with MDD. MI and daily function difficulties disrupt the ability to manage daily routine, causing impairment in occupational balance (OB) and affecting the resilience and quality of life (QoL) of people with MDD.

**Objectives:**

A preliminary cross-sectional study was conducted to advance the understanding of MI and its relationships to daily function, OB, and QoL in people with MDD.

**Methods:**

**“MOBILE” Model Development:** Data was collected using various assessments, utilized by Ecological Momentary Assessment (EMA), zooming in on real-life patterns of daily lives of people with MDD. Based on the research findings, a theoretical model, the **Mood-Occupation Balance Reciprocal Model (MOBILE),** was constructed, presenting the relationships between the variables.

**Results:**

This presentation will showcase the research findings and the reciprocal relationships between study variables, which form the foundation for this model development.

**Conclusions:**

The “Mobile” model addresses the two core components of depression - MI and functional impairment, and explores their relationships to QoL, OB, participation, and personal resilience in people with MDD. The model serves as a platform for building a personalized clinical intervention, which will be tested in further research.

**Implications for Future Practice:**

Currently, most treatments offered for MDD combine pharmacological and psychosocial treatments. Yet, their efficacy in improving functional abilities is limited. The development of the current model, and subsequently the development of a clinical intervention, allows for the expansion of treatment options for people with MDD. In this way, it may assist in reducing the heavy burden of the disease, improve functioning, and prevent recurrence. Additionally, the use of EMA allows for the efficient and accurate collection of data, thereby extending the diagnostic and treatment processes from the clinic to everyday environments.

**Disclosure of Interest:**

None Declared

